# Navigating Serious Illness: The Role of Palliative Care Consultations in Nursing Homes

**DOI:** 10.1093/geroni/igaf122.153

**Published:** 2025-12-31

**Authors:** Elisha Oduro, Ruth Palan Lopez, Kathleen Unroe, Alexandra Mora, Moses Akwobugi, Joan Carpenter

**Affiliations:** University of Maryland Baltimore, Baltimore, Maryland, United States; Massachusetts General Hospital Institute of Health Professions, Boston, Massachusetts, United States; Indiana University School of Medicine, Indianapolis, Indiana, United States; University of Maryland Baltimore, Baltimore, Maryland, United States; University of Maryland Baltimore, Baltimore, Maryland, United States; University of Maryland School of Nursing, Baltimore, Maryland, United States

## Abstract

Palliative care (PC) consultations in nursing homes (NHs) are recognized for enhancing the quality of life for residents living with serious illness. Despite their benefits, the accessibility of PC consultations remains variable and inconsistent across NHs. This study aimed to develop a framework to explain PC consultation implementation and utilization in NHs from the perspective of PC and NH key partners. A constructivist grounded theory approach was employed, using semi-structured and group interviews with PC and NH administrators, nursing directors, social workers, nurses, primary care providers, and PC specialist nurse practitioners. Participants (N = 33) were purposively sampled from three PC agencies with relationships in 14 NHs to capture variability in PC implementation and utilization. Data were analyzed through open, process, and theoretical coding to develop a theoretical framework. The central issue influencing PC consultations in NHs was the need to navigate resident and family uncertainty and complexity in serious illness care, especially for residents living with dementia. NH staff used PC consultations to guide residents and families during unpredictable disease trajectories, mediate family and institutional expectations by “bridging the gap”, and reframe PC as integrated support rather than only for end-of-life care. PC consultations facilitated better sensemaking, improved communication, and led to more effective care planning. PC consultations serve as strategic tools for NH staff to manage the complexities of serious illness care. Understanding and supporting the practical applications of PC consultations within NHs can lead to more effective integration and utilization of these services, ultimately enhancing resident care.

